# The Role of Gender in the Employment, Career Perception and Research Performance of Recent PhD Graduates from Dutch Universities

**DOI:** 10.1371/journal.pone.0164784

**Published:** 2016-10-13

**Authors:** Cathelijn J. F. Waaijer, Hans Sonneveld, Simone E. Buitendijk, Cornelis A. van Bochove, Inge C. M. van der Weijden

**Affiliations:** 1 Centre for Science and Technology Studies, Faculty of Social and Behavioural Sciences, Leiden University, Leiden, The Netherlands; 2 Netherlands Centre for Graduate and Research Schools, Amsterdam, The Netherlands; 3 Tilburg Law School, Tilburg University, Tilburg, The Netherlands; 4 Leiden University Medical Center, Leiden, the Netherlands; Universitat Jaume I, SPAIN

## Abstract

Recent decades have seen a sharp increase in the number of female PhD graduates in the Netherlands. Currently, the share of females among newly graduated PhDs is almost on par with that of males. A considerable body of scientific studies has investigated the role of gender in the academic workplace. However, the role of gender in the careers of all PhD graduates, including those outside academia, has been studied less. In this study, we investigate gender differences in type of job, occupation, career perception and research performance of recent PhDs. The study is based on a survey of persons who obtained a PhD from one of five Dutch universities between 2008 and early 2012. We show that gender differences in post-PhD careers are non-existent in some aspects studied, but there are small differences in other aspects, such as sector of employment, type of contract, involvement in teaching and management, and career perception. In contrast, male and female PhDs differ sharply on two factors. The first is field of PhD, females being heavily underrepresented in engineering and the natural sciences. The second is part-time employment, females being much more likely to work part-time than males, especially if they work in the Netherlands. In later career stages, the combination of the small and large differences can be presumed to affect the career progression of female PhDs through cumulative disadvantage.

## Introduction

The pool of highly educated women (with a master’s or doctoral degree) is larger than ever. In many countries, numbers of female PhD graduates have increased much more than numbers of male PhDs over the past decades, women receiving 47 per cent of 2012 doctoral degrees in the European Union [1]. The Netherlands is no exception to this trend [2, 3]. This raises the question whether the career interests of and opportunities for female PhDs follow the same trend towards gender equality as the percentage of PhDs. Although a sizeable body of scientific literature on the role of gender on academic careers exists, the topic of gender differences in the post-PhD careers of *all* PhDs, including those outside academic research, remains underexplored. In this study, we provide evidence on gender differences in job type, occupation, career perception and research performance of recent PhDs from Dutch universities.

First, we will give a short overview of the existing literature on gender and (academic) careers. Women are still heavily underrepresented in higher positions, both in academia and in other sectors [4–6]. One explanation for this is the pipeline argument, which says that when the number of women among entrants rises, so will the share of women in top positions. In science, technology, engineering and mathematics (STEM), this certainly holds true, as females are heavily underrepresented in these fields, for example in the United States, Canada and the Netherlands [7–9]. However, the pipeline argument alone cannot explain the underrepresentation of women in top positions in academia and business [5]. For the underrepresentation of women in top academic positions, many other explanations have been proposed, including but not limited to gender differences in career interest, differences in performance and (implicit) gender bias in hiring, promotion and research funding decisions. In many cases, gender differences are quite small, but over time these differences lead to a cumulative disadvantage for female academics [10].

Job activities differ by gender: female faculty are more involved in teaching, whereas their male counterparts are more likely to focus on research [11, 12]. This may also affect promotion decisions, as these are often based on research performance [13, 14]. Research production also differs, with female scientists lagging behind their male counterparts [15, 16]. However, this result is obtained without a correction for differences in hours worked. The stage of the career may be important, as young social scientists in the Netherlands do not show gender differences in production [17]. In contrast, in the same group ten years later, the total productivity of males was higher than that of females, suggesting that whereas production is similar for male and female academics in early career stages, in later career stages gender differences occur [18]. When it comes to citation impact, most studies find small or no differences between male and female scientists [16, 18, 19].

There is also evidence for gender bias in hiring, promotion and funding decisions. Female academics are less likely to be hired or promoted than male academics, even with the same job experience and accomplishments [20–23]. In hiring processes in the Netherlands, gender also plays a role [24]. However, the evidence is conflicting, others finding no influence of gender in career progression [25, 26]. When it comes to the role of gender in research funding, the scientific literature is also divided. Based on a large review of the literature Ceci and Williams [27] argue that gender differences in funding are small, or even non-existent. In contrast, others find that female scientists are in a disadvantaged position, with women receiving less funding in total [16], receiving smaller grants [4], and having a smaller chance of success when submitting a grant proposal [28]. In the Netherlands a recent study found gender bias in the allocation of grants from the most important national person-oriented research funding schemes [29]. However, this finding was later disputed [30].

As such, there is quite some literature on gender differences among academic researchers, but less is known about gender differences in the post-PhD careers of all PhDs. There is some evidence on the subject: in the Netherlands, females are less likely to work as a researcher than males, are also less likely to work in the business enterprise sector, but more likely to work in the private non-profit sector [31]. In the U.S., too, females are less likely to work in the business sector; there they are more likely to work in the academic sector [32]. These breakdowns, however, do not take into account time since PhD. Since the share of male PhDs was much higher in the past, lumping PhDs of several cohorts together may sharply bias findings. A study of a more homogeneous group of recent PhDs in Denmark found no effect of gender on the sector of employment [33]. The activities performed in PhDs’ work also differ between women and men, with female PhDs in Australia being more involved in teaching and advising or mentoring students and male PhDs being more involved in research, supervising or management, product development and the commercialization of research products [34]. Earlier, Fox and Stephan [35] found that female doctoral students at U.S. universities are more interested in academic teaching than their male counterparts.

With respect to type of contract, female PhDs from Australian universities are slightly more likely to have a temporary contract [34]. For the Netherlands, Sonneveld et al. [36] found that females are more likely to have a temporary contract when working outside academia. Female PhDs are employed part-time much more often than males, especially in Germany, Belgium and the Netherlands [37]. Nevertheless, in the Netherlands part-time employment among female PhDs is much lower than among female master graduates, female PhDs working on average almost four hours per week more than female master graduates [37, 38]. These gender differences also raise the question whether male and female PhDs perceive their career prospects differently. In the United States, Fox and Stephan [35] found that male PhD students were more positive about their career prospects in industry or government than female PhD students, whereas females were more positive about career prospects in academic teaching.

The Netherlands was chosen as a country of study as the share of females among PhD graduates is comparable to the EU-28 average and trends in the growth of PhD graduates, both male and female, also mirror the EU-28 average [1]. At the same time, from student to full professor, Dutch universities have a gender distribution reminiscent of a scissor: an almost equal gender distribution among students, a slight *overrepresentation* of women among the bachelor and master graduates, but after that, the share of women drops from 44% among PhD candidates to 17% among full professors [6]. This pattern also occurs among the higher educated in general, with women in the 25–45 age bracket being higher educated than men. In addition, in many sectors the share of women in occupations requiring higher education is greater than the share of men [39]. However, the higher the position in an organization, the lower the share of women. This is not simply the result of underrepresentation of women when the current top managers started their careers–although the share of women has risen slightly over the years, the gap between the share of women in the top management level and lower levels has remained quite stable. Therefore, the pipeline argument cannot explain the gender differences. One of the explanations given for the scissor pattern in gender distribution is that many women in the Netherlands, also the higher educated, work part-time, which could limit their career progression [40]. As discussed at the start of the introduction, timing may play an important role in the occurrence of any gender differences in careers, and the pipeline argument may explain some. Using a sample of recent PhDs makes it possible to study whether gender differences already occur quickly after the conferral of the PhD degree.

In this study, we delve further into the role of gender in post-PhD careers, by investigating gender differences in the careers of recent PhDs from five Dutch universities.

We address the following research questions:

1. Does the type of job (sector of employment, level of job, and type of contract) of PhDs differ by gender?
2. Does the occupation of PhDs differ by gender?
3. Does the perception of career prospects differ by gender?
4. Does the (perception of) research performance differ by gender, i.e., do male and female PhDs receive research funding to the same extent, and do they perceive their scientific oeuvre differently?

When gender differences were found, we also investigated through which mechanisms these differences could have occurred by controlling for factors that have been identified as influencing factors in the scientific literature.

## Data and Methods

Below, we provide a summary of the survey methodology and measured variables. A more elaborate description of the survey questionnaire, methodology and variables is given in a working paper [41].

The *survey sample* consisted of 2,193 PhD graduates who obtained a PhD from Utrecht University (a broad research university), Delft University of Technology (engineering and technology), Wageningen University (an agricultural university), or Erasmus University Rotterdam (focused on medicine and social sciences, especially economics and management) between April 2008 and March 2009 or from Leiden University (a broad research university) between January 2008 and May 2012. An invitation to the survey (which was open from 23 October 2013 until 21 January 2014) was sent through email or LinkedIn, in which the prospective respondents were informed on the purpose and content of the survey in the invitation, and strict confidentiality guaranteed, only aggregate results (impossible to trace back to individuals) to be published. Furthermore, a test of the survey showed the survey took 20 minutes to complete on average, which was also written in invitation letter, so the respondents would know which response burden to expect. In the online survey itself, the instructions made explicit it was possible to quit the survey. Up to three reminders were sent if respondents had not completed the survey. In total, 1,133 started the survey (52%), and 960 progressed to the final question (44%). Survey data were anonymized before analysis and the key to the respondents’ names and unique survey analysis ID stored in a secured folder.

Non-response analysis showed that the respondents were representative of the survey set regarding gender, age, year of PhD, and city of PhD [41]. However, Dutch nationals seemed to be overrepresented in the survey compared to the country of birth of the entire sample.

In this study, we used variables on type of job, perception of career prospects, research performance and personal characteristics. Three *sectors of employment* were distinguished: academic R&D (dubbed academia in the paper for brevity), non-academic R&D (dubbed non-academic research) and non-R&D (dubbed outside research). The classification of respondents into these categories was based on two variables: involvement in R&D and type of employer. We follow the Organisation for Economic Co-operation and Development’s (OECD) typology of R&D: basic research, applied research, and experimental development [42]. PhDs not involved in any of the three in their main job were classified as working in non-R&D (further dubbed outside research). PhDs in academic R&D (further dubbed academia) are PhDs involved in R&D and employed at a university, university of applied sciences or college, academic hospital, or research institute. PhDs in non-academic R&D (further dubbed non-academic research) are PhDs involved in any type of R&D and working at another type of institution (e.g., at a private business [incl. an own business], government institution, non-academic hospital). Furthermore, respondents were asked whether they were working in or outside the Netherlands.

The *level* of the respondents’ job was also determined, through two multiple-choice questions. Two aspects of job level were determined: whether respondents had a supervisory role, and the education level normally required for their job. The four education levels were bachelor or lower, master, PhD, and professional degree (e.g., medical degree). For the respondents with a job, we measured which *type of contract* they had. We distinguish five types of employment (contract): permanent contract, probation period of a permanent contract, tenure track contract, temporary contract without prospect of permanence, and self-employment. Furthermore, we asked employees whether they were employed full-time or part-time. Full-time was regarded as working the maximum number of hours possible according to the sector’s collective labor agreement; part-time as less than this maximum. This choice was made because we expect a large share of PhDs work in environments where full-time employment is the norm [14]. Here, we deviate from the standard Dutch classification that considers part-time employment as employment for fewer than 35 hours per week [43]. However, this standard classification was mainly constructed in this way because the number of hours constituting full-time employment differs by sector. By asking respondents whether they work full-time according to their sector’s collective labor agreement, we solve this problem. Furthermore, we developed a *classification of PhDs’ occupations*.

*Perception of career prospects* was determined by asking respondents how they would rate “long-term career perspectives” and “the availability of permanent positions” in academia, non-academic research and outside research on a five-point Likert scale ranging from “very bad” to “very good”. Furthermore, a few aspects of the PhDs’ research performance were measured. We asked respondents whether they had *received a personal grant* for doing research. Respondents were also asked to rate their *perception of their own scientific oeuvre*, by indicating to which extent they agreed with the following statement: “my scientific oeuvre is good enough to build an academic research career on” (on a four-point scale).

In the survey, we also asked the respondents for their *gender* (female or male, with an explicit answer option in the survey not to tick one of the two). *Nationality* was measured as a dummy for high income OECD countries. The variable measures whether a PhD has the nationality from one of 21 OECD countries with a per capita Gross Domestic Product greater than $32,000 Purchasing Power Parity in 2012 [44]. These countries are Australia, Austria, Belgium, Canada, Denmark, Finland, France, Germany, Iceland, Ireland, Italy, Japan, Luxembourg, the Netherlands, New Zealand, Norway, Spain, Sweden, Switzerland, the United Kingdom of Great Britain and Northern Ireland, and the United States of America.

We also measured the respondents’ *age at the time of the survey*, whether they were *living with a partner*, and whether they had *children below the age of six*. In addition, we determined the *field of PhD* (medical and health sciences, natural sciences, social sciences, humanities, or engineering and technology) and *years since PhD*.

## Results

### Type of job

First, we looked at the type of job that PhDs had, and assessed whether there were gender differences. One aspect of job type is the *sector of employment*. Female PhDs were more likely than their male counterparts to work in academia (64% of females vs. 56% of males), whereas males were more likely to work in non-academic research (34% of males vs. 22% of females; *p* < 0.001 in Pearson’s χ2 test of independence). A simple explanation for this fact may be that in fields where females are traditionally underrepresented (i.e., natural sciences, and engineering and technology), more PhDs will go into non-academic research. Among recent PhDs in the Netherlands, women are also underrepresented in engineering and technology (22% of PhDs in this field are female) and in the natural sciences (39% female). On the other hand, there is gender parity in the medical and health sciences (54% female), social sciences (52%) and humanities (47%). To assess the effects of gender distribution by field on sector of employment, we calculated what the sector of employment of male and female PhDs would have been if the gender distribution in all separate fields would have been the same as in the entire group of respondents. This decreased the gender differences slightly, but women were still more likely to work in academia and men more likely to work in non-academic research.

Second, we analyzed the *level* of the PhDs’ job. Three aspects of job level were measured: the educational level normally required for the job and whether the PhD has a supervisory role. Over half of the respondents had a supervisory role in their jobs. Most worked at PhD level, but there was a considerable group working below this level of educational attainment: close to a quarter, a share that is slightly higher than the overall share of persons in the Netherlands working below their level of educational attainment, which was 20% in 2011 [45]. Our findings suggest that a sizeable share of recent PhDs is overeducated for their job, and in another article we show that this overeducation has a considerable negative effect on job satisfaction [46]. This effect occurs for comparable numbers of male and female PhDs, though, as there were no statistically significant differences in job level between female and male PhDs. This shows that at early career stages, gender does not influence the level of the job.

Third, we analyzed whether gender affects the *type of contract* of recent PhDs. We found no large differences between the percentages of females and males in a probation period (both 3%), on a tenure track contract (7 vs. 6%) or being self-employed (6 vs. 8%). However, there were large differences between females and males regarding permanent employment and temporary employment without prospect of permanence: a smaller share of females was employed on a permanent contract (45%, vs. 55% of the males; *p* = 0.002 in Pearson’s χ2 test of independence), and a larger share on a temporary contract without prospect of permanence (39%, vs. 29% of the males; *p* < 0.001).

It may be that other factors underlie this difference, such as sector of employment (women are more prone to work in academia, where temporary contracts are more prevalent) or field of PhD (men are more likely to do a PhD in engineering and technology, where employers may offer job security to be an attractive employer to scarce talent). Therefore, we performed a logistic regression for temporary employment without prospect for permanence on gender, other personal characteristics, sector of employment, time since PhD, and the field of PhD. As the effects of having children may be different for males and females, we also included an interaction term between gender and children.

The results show that after controlling for these other factors, gender did not influence the likelihood to have a temporary contract without prospect of permanence (Table 1). Having children below the age of six was associated with a smaller likelihood. Although there was a slight interaction effect between gender and children, this effect was not statistically significant. Instead, the sector of employment did influence this likelihood, as outside academia a temporary contract was much less likely than in academia. Time since PhD was also an important factor: the longer it was, the smaller the likelihood of a temporary contract. Unsurprisingly, older PhDs were less likely to have a temporary contract. Furthermore, PhDs from the medical and natural sciences, and the humanities, were more likely to have a temporary contract than engineering PhDs. Thus, it is through sector of employment, field of PhD and personal characteristics that a larger percentage of females had a temporary contract than males. As such, direct bias against female PhDs in contract negotiations does not seem to occur. However, it is important to keep in mind that although gender does not *independently* affect the odds to have a temporary contract, from an explanatory perspective it can still play a role. For example, feminization of an profession often leads to a penalty on the wages in that profession [47]. It may be that in fields with many women, such as the social sciences, temporary employment is customary *exactly* because they have a large share of women. Thereby, gender could still play a role, but not have an independent effect in a regression model.

**Table 1:** Effect of several employment, personal and PhD characteristics on the employment on a temporary contract without prospect of permanence.

	B (S. E.)	p-value
Intercept	2.92 (0.90)	0.001**
Female	0.03 (0.25)	0.902
Children below 6	-0.69 (0.28)	0.014*
Female x children below 6	0.66 (0.38)	0.083
Nationality of high-income OECD country	0.46 (0.32)	0.158
Living with partner	-0.17 (0.24)	0.475
Age at survey	-0.09 (0.02)	< 0.001***
Years since PhD	-0.21 (0.08)	0.007**
*Sector of employment (ref*. *is academia)*		
Non-academic research	-2.02 (0.27)	< 0.001***
Outside research	-1.01 (0.29)	< 0.001***
*Field (ref*. *is engineering and technology)*		
Medical and health sciences	1.27 (0.42)	0.002**
Natural sciences	0.92 (0.42)	0.028*
Social sciences	0.18 (0.46)	0.705
Humanities	1.31 (0.47)	0.005**

*, **, and *** denote statistically significant difference of the independent variable at the 5, 1, and 0.1% level, respectively. Analysis based on 657 observations.

We also looked at *part-time employment*. On the whole, a much larger share of female PhDs was employed part-time (Table 2; 34% of females vs. 12% of males). The sector with the largest share of part-time employment was “Outside research”, followed by non-academic research and academia. Of male PhDs outside research, too, a relatively large percentage worked part-time. Among female PhDs, part-time employment was especially common for those with young children: 52% of females with children below the age of six worked part-time compared to 23% of women without young children. For males, these percentages were 15% for those with children below six and 11% without young children. In addition, working part-time was much more common in the Netherlands than outside it: 31% of the PhDs in the survey working in the Netherlands worked part-time, compared to just 6% of those working outside the Netherlands. This high figure is mainly due to female PhDs: 47% of females in the Netherlands worked part-time, compared to 17% of males.

**Table 2:** % of employees working part-time, by sector of employment and gender.

	Male	Female	Total
		*%*	
Academia	10	31	20
Non-academic research	12	43	23
Outside research	26	41	34
Total	12	34	22

### Occupation

So what did PhDs actually do in their jobs, i.e., what is their *occupation*? In official statistics, the classification of occupations that is used is the International Standard Classification of Occupations (ISCO-08) from the International Labour Organization [48]. In this classification, most PhDs are classified into the main categories “professionals” and “managers”. Further sub classifications are by field, e.g., science and engineering professionals, teaching professionals, etcetera. Unfortunately, this may be problematic for scientists who are involved in multiple activities, such as university research and teaching (which are two categories in ISCO-08). The same problem occurs when PhDs are involved in both consulting and teaching, or any other combination of activities that are grouped in different occupational categories. However, for PhDs it is not sufficiently fine-grained. Therefore, we developed our own classification of PhDs’ occupations:

1. PhDs active in education (subdivided into non-academic level, at an institution for higher vocational education or at university level).
2. PhDs active in research (subdivided according to job level into junior [postdoctoral researchers, junior scientists and research assistants], senior [associate professors, full professors, senior scientists] and intermediate [associate professors, researchers (without prefix or suffix) and all other job titles].
3. Content specialists: professionals who do not perform research or teach, but use the knowledge they obtained during their educational training in their job (subdivided into consultants, policy advisors, four health care categories, lawyers and other legal professionals, and other content specialists).
4. PhDs active in management (subdivided into research management, general management and self-employed).
5. PhDs in other occupations.

In this classification, PhDs can be classified into multiple major categories, but only one sub category is possible. Examples of professions in each category are given in Table 3. The respondents were classified into these categories on the basis of their answers to two open questions, the first asking what the job title of their main job was, the second asking what the respondents did in their main job.

**Table 3:** Classification of occupations and examples.

Category	Example
Education	
Non-academic	High school teacher
Higher vocational education	Lector
University	Assistant professor
Research	
Junior	Postdoctoral researcher
Intermediate	Group leader (in research), assistant professor
Senior	Associate professor, full professor, senior scientist
Content specialist / consultant	
Consultant	Strategic consultant
Policy advisor	Policy advisor
Medical specialist	Cardiologist
Clinical fellow	Doctor in training to become a medical specialist
Medical specialist and clinical fellow	Neurologist also training in pathology*
Other health care	Clinical psychologist
Lawyers and other legal professionals	Lawyer
Other content specialist	Data analyst, technology specialist
Manager	
Research manager	Project manager of European projects
General manager	Technical project manager
Self-employed	Partner in start-up company
Other	Carpenter*

* Fictitious label to prevent identification of individuals.

One third of recent PhDs from Dutch universities was active in education, of which most were involved in university teaching (Table 4). Seven in ten were involved in research or experimental development according to the open answers. Hence, there is a discrepancy between the respondents’ answers to multiple-choice questions that showed 88% were active in research and development, and their answers to an open question. This slight discrepancy is probably due to the fact that we asked respondents whether they did *any* R&D in the multiple-choice questions, whereas respondents may focus on their main job activities in answering an open question. Four in ten PhDs worked as content specialists, of which 30% as a medical specialist or as fellow training to become one. The group of content specialist also contains a considerable number of consultants, policy advisors and legal professionals. Furthermore, many PhDs in this group were working as “other” content specialists, e.g., as museum curator, clinical research associate or at a publisher. Finally, almost three in ten PhDs had a management job, of which most in research management.

**Table 4:** Job activities by gender (multiple main categories possible).

	Male	Female	Total
		*%*	
Education	29	38	33**
*of which*:			
Non-academic	2	2	2
Higher vocational	4	3	4
University	93	95	94
Research	71	71	71
*of which*:			
Junior	22	29	25
Intermediate	53	53	53
High	24	19	22
Content specialist	39	38	39
*of which*:			
Consultant	16	13	15
Policy advisor	4	7	5
Medical specialist	19	15	18
Clinical fellow	10	14	12
Both medical specialist and clinical fellow	0	< 1	< 1
Other health care	4	7	5
Lawyers and other legal professionals	4	1	3
Other content specialist	43	42	43
Management	25	31	28*
*of which*:			
Research management	62	71	67
General management	36	27	31
Self-employed	2	2	2
Other	< 1	< 1	< 1

* and ** denote statistically significant difference at the 5 and 1% level, respectively.

Gender differences were only found in the main categories of education and management. Female PhDs were more likely to be involved in education, a finding also obtained in other studies [34, 35]. However, females were underrepresented in the natural sciences, and engineering and technology, which have lower teaching loads than other fields [49]. It may be that field of PhD is actually mediating the gender differences. Therefore, we again calculated what would have happened if the gender distribution would have been the same in all fields. Once more, gender differences became slightly smaller, which shows that the underrepresentation of females in some fields explains a part of the differences between female and male PhDs. However, females still had a statistically significantly higher chance of being involved in education.

In contrast to Maas et al. [31] and Dever et al. [34], we found no gender difference in PhDs’ involvement in research, and found that female PhDs were actually *more* likely to be involved in management, whereas Dever et al. found that male PhDs were more likely to be involved in management. [31]Here, overrepresentation or underrepresentation of women per field of PhD did not affect the gender differences. Especially in light of Maas et al’s study [31], it might seem surprising that we found no gender differences in PhDs’ involvement in research, as they also studied PhDs in the Netherlands and found male PhDs to be working as researchers more often. However, their study included PhDs who got their degree between 1990 and 2013, but did not control for year of PhD. In earlier years, the share of men was much higher than now. This is likely to have influenced their results. In comparison, the PhDs in our study are a much more homogeneous group.

Despite the lack of gender differences in research involvement, the fact that female PhDs were more likely to be involved in education may mean that in academia, female PhDs have a higher teaching load than male PhDs. Of the PhDs involved in research or teaching, we analyzed which share of PhDs was involved in both teaching and research, which share only in research and which share only in teaching. Female PhDs more often combined teaching and research (52% of females compared to 47% of males), whereas male PhDs were more often involved in research only (51% of males compared to 46% of females). However, these differences were small and not statistically significant.

For the PhDs with research position in their job description, we also analyzed the *level* of their position, i.e., junior, intermediate and senior. This analysis showed that male PhDs were more likely to have a senior researcher position, and female PhDs to have a junior position, but these differences were not statistically significant (Table 4).

### Perception of career prospects

In the survey, respondents were asked to *rate* several aspects of *career prospects* in academia, non-academic research and outside research (on a five-point Likert scale ranging from “very bad” to “very good”). Here, we will highlight two: long-term career perspectives and the availability of permanent positions. We compared the perceptions of females and males of these two aspects in the three aforementioned sectors. As the data were not normally distributed, we performed a Mann-Whitney U test to test whether the differences between the groups were statistically significant. We found that females were more negative about both the long-term career perspectives and the availability of permanent positions, in all three sectors (Table 5). These differences were statistically significant in all cases except for the perception of long-term career perspectives outside research.

**Table 5:** Differences in perception of career prospects by gender (Mann-Whitney U test).

Sector	Career aspect	Mean rank females (N females)	Mean rank males (N males)	p-value
Academia	Long-term career perspectives	405.05 (390)	449.88 (468)	0.007**
	Availability of permanent positions	400.84 (397)	458.55 (466)	< 0.001***
Non-academic research	Long-term career perspectives	342.43 (323)	385.50 (409)	0.004**
	Availability of permanent positions	323.15 (319)	382.73 (392)	< 0.001***
Outside research	Long-term career perspectives	337.31 (302)	357.08 (394)	0.171
	Availability of permanent positions	317.47 (304)	358.26 (375)	0.005**

** and *** denote statistically significant difference at the 1 and 0.1% level, respectively.

We hypothesized that besides gender, nationality, age and field of PhD could influence the perception of career prospects. Nationality was measured as a dummy for high income OECD countries. This was done because researchers from lower income countries may decide to obtain a PhD in the Netherlands to increase their career opportunities in their home country. As such, PhDs from lower income countries may rate their career prospects with the home country in mind, and perceive them as better than they would rate career prospects in high-income countries. Indeed, Stephan et al. [50] found that increasing career prospects in the home country is an important reason for researchers to do a PhD abroad.

We performed an ordinal logistic regression with the perception of career prospects (five-point Likert scale) as the dependent variable (Table 6). Our results show that gender independently influenced the perception of the availability of permanent positions in all three sectors: females rated this availability as worse than males. Furthermore, females rated the long-term career perspectives in academia as worse. However, another important explanatory factor was nationality: PhDs from high-income countries were more negative about both long-term career perspectives and the availability of permanent positions in academia and non-academic research.

**Table 6:** Effect of gender, nationality, age at survey and field of PhD on the perception of long-term career perspectives and the availability of permanent positions in academia, non-academic research and outside research (by ordinal regression).

	Academia			Non-academic research		Outside research	
Long-term career perspectives	B (S. E.)		p-value	B (S. E.)	p-value	B (S. E.)	p-value
3Female	-0.36 (0.14)		0.012*	-0.30 (0.16)	0.057	-0.08 (0.16)	0.622
Nationality of high-income OECD country	-1.57 (0.24)		< 0.001***	-0.85 (0.26)	0.001**	0.15 (0.28)	0.598
Age at survey	0.03 (0.01)		0.024*	0.01 (0.01)	0.238	0.00 (0.01)	0.997
*Field (ref*. *is engineering and technology)*							
Medical and health sciences	0.04 (0.25)		0.867	-1.03 (0.27)	< 0.001***	-0.94 (0.27)	< 0.001***
Natural sciences	-0.27 (0.25)		0.293	-0.74 (0.27)	0.006**	-0.39 (0.28)	0.153
Social sciences	0.28 (0.28)		0.317	-1.31 (0.31)	< 0.001***	-0.92 (0.31)	0.003**
Humanities	-0.16 (0.30)		0.599	-2.25 (0.34)	< 0.001***	-1.68 (0.34)	< 0.001***
*Number of observations*	*684*			*592*		*567*	
**Availability of permanent positions**							
Female	-0.54 (0.15)		< 0.001***	-0.42 (0.16)	0.009**	-0.34 (0.16)	0.036*
Nationality of high-income OECD country	-1.42 (0.23)		< 0.001***	-0.52 (0.26)	0.043*	-0.08 (0.27)	< 0.001***
Age at survey	0.49 (0.12)		< 0.001***	0.00 (0.01)	0.834	-0.02 (0.01)	0.079
*Field (ref*. *is engineering and technology)*							
Medical and health sciences	-0.11 (0.25)		0.652	-0.84 (0.26)	0.001**	-1.24 (0.27)	< 0.001***
Natural sciences	-0.50 (0.26)		0.051	-0.48 (0.27)	0.074	-0.46 (0.28)	0.097
Social sciences	0.13 (0.29)		0.640	-1.31 (0.31)	< 0.001***	-1.11 (0.31)	< 0.001***
Humanities	-0.46 (0.31)		0.138	-1.67 (0.34)	< 0.001***	-1.66 (0.34)	< 0.001***
*Number of observations*	*687*			*567*		*549*	

*, **, and *** denote statistically significant difference of the independent variable at the 5, 1, and 0.1% level, respectively.

### Research performance

As described in our literature review, women tend to receive less research funding than men. To get an idea about whether there were gender differences in *research funding* among our respondents, we asked them whether they had received a grant for doing research. A total of four out of ten out of all PhDs had, with women actually being more likely to have received one: 45% of female PhDs had, compared to 37% of males (*p* = 0.006 in Pearson’s χ2 test of independence). However, as indicated in the first part of the results section, a greater share of women than men were working in academia. Among only those currently working in academia, 54% of females had received a grant, compared to 48% of males, but this result was not statistically significant (*p* = 0.138). Clearly, female and male recent PhDs from Dutch universities are equally likely to obtain research funding.

One explanation given for gender gaps in academia is that women are less confident about their capabilities and careers than men [51]. On the other hand, studies on the academic productivity of male and female academics also suggest the gender gap in publishing may be closing [17]. Therefore, we asked the respondents how they would rate their *scientific oeuvre*. A slightly higher share of men indicated that their scientific oeuvre is “more than good enough” to build an academic career on (22% of males vs. 16% of females). However, a slightly higher share of women said their scientific oeuvre is “good enough” (56% of females vs. 52% of males). Furthermore, neither of these differences were statistically significant. Therefore, among recent PhDs in the Netherlands, women are as confident about their scientific oeuvre as their male counterparts.

## Discussion and Policy Implications

When assessing gender differences in the employment situation, career perception and research performance of recent PhDs from Dutch universities, the most striking finding is that for most characteristics the differences between female and male PhDs are only small. They mainly pertain to sector of employment, type of contract and occupation, in the latter case only in involvement in teaching and management. By themselves, these differences are not very meaningful and the lack of real differences in these aspects is encouraging from the perspective of gender equality. However, taken together and combined with the large gender difference in part-time employment, they could lead to larger differences later in the career: “many mole hills together become a large mountain” [52]. Previous studies have shown that small differences together lead to larger differences in later career stages, through cumulative disadvantage [10, 52]. Suggestively, female PhDs in our study were more negative about their career prospects than male PhDs. In the literature, gender disparities such as the ones found in our study, are partly ascribed to culture-specific national perception of femininity and masculinity in relation to science, work and family, as well as to a culture-specific masculinist model of science, including male-oriented organizational, social and cultures norms within the academic working environment [14, 53].

There already is a large gender difference before women and men even embark on a PhD, namely the choice of field of study. In most industrialized countries, women now make up over fifty per cent of all university students, but they are still underrepresented in the STEM fields, for example in Canada and the United States [7, 8]. The Netherlands is no exception, with female students being overrepresented in education and social sciences, but heavily underrepresented in the natural sciences, and engineering and technology in 2013/’14 [9]. Several explanations have been given for this phenomenon; Blickenstaff [54] outlines nine, including but not limited to attitude and early experiences, curriculum design, teachers’ attitude towards boys and girls, and the pressure to fulfill gender roles. Gender stereotypes with respect to science are still pervasive: science is associated with men, and this association is especially strong in the Netherlands [55]. In the Netherlands, the gender differences at high school level seem to be decreasing though, with more female high school students now following a curriculum oriented towards the natural sciences and engineering (an increase from 20% in 2007/’08 to 38% in 2013/’14; [56]). This suggests that, in time, the share of females among PhDs in the natural sciences and engineering may also increase, but that the share of females will still lag behind that of males for a considerable number of years.

The second large gender difference we found was in part-time employment, with female PhDs working part-time much more often than male PhDs. In itself this is not a surprising finding, as part-time employment is very common among women in the Netherlands, more so than in any other OECD country [57]. An explanation for this phenomenon could be found in the fact that traditional motherhood ideology is still surprisingly strong in the Netherlands [40]. In addition, part-time employment has become institutionalized, especially for women and even for high-skilled work, which enables such a large share of women to work part-time [58]. However, the fact that female PhDs work part-time more often (especially those with young children, and especially those working in the Netherlands) may hamper their career advancement in the long run, as the model of the ideal worker still includes full-time employment [14, 51, 59]. Indeed, although differences in job level of PhDs in research were very small in our study, men were more likely to occupy a senior position. This may be why female PhDs are less positive about academic career prospects, despite being as confident about their scientific oeuvre as men.

In conclusion, there are only small gender differences in the job type, occupation, career perception and research performance of recent PhDs from Dutch universities. However, through accumulation these small differences and the large differences in field of study and part-time employment, can lead to more serious gender gaps in later career stages, both in academia and in other sectors of employment.

## References

[1] European Commission. She Figures 2015: Gender in Research and Innovation Luxembourg: Publications Office of the European Union; 2015.

[2] De Goede M, Belder R, De Jonge J. Promoveren in Nederland: Motivatie en loopbaanverwachtingen van promovendi The Hague: Rathenau Instituut; 2014.

[3] Statistics Netherlands. Aantal gepromoveerde vrouwen neemt toe, vooral in alfa- en gamma-richting. 2014 Dec 5 [cited 21 October 2015]. Available: http://f.cbs.nl/nl-NL/menu/themas/arbeid-sociale-zekerheid/publicaties/artikelen/archief/2014/2014-4185-ta.htm.

[4] Shen H. Mind the Gender Gap. Nature. 2013;495(7439): 22–4. 10.1038/495022a 23467149

[5] Hoobler JM, Lemmon G, Wayne SJ. Women's underrepresentation in upper management: New insights on a persistent problem. Organ Dyn. 2011;40(3): 151–6. 10.1016/j.orgdyn.2011.04.001

[6] Landelijk Netwerk Vrouwelijke Hoogleraren. Hoogleraren. Monitor Vrouwelijke Hoogleraren 2015. Ede: Landelijk Netwerk Vrouwelijke Hoogleraren; 2015.

[7] National Science Foundation, National Center for Science and Engineering Statistics. Women, Minorities, and Persons with Disabilities in Science and Engineering: 2015. Special Report NSF 15–311. Arlington: National Science Foundation/National Center for Science and Engineering Statistics; 2015.

[8] Hango D. Gender differences in science, technology, engineering, mathematics and computer science (STEM) programs at university Ottawa: Statistics Canada; 2013.

[9] CBS Statline—Hoger onderwijs; ingeschrevenen, studierichting, leeftijd. 2015 7 Aug [cited 29 October 2015]. Available: http://statline.cbs.nl/Statweb/publication/?DM=SLNL&PA=70943ned&D1=0&D2=a&D3=0&D4=1&D5=1&D6=0-1,42,88,149,181,217,233,273,300-303&D7=23&HDR=T,G2,G4,G5&STB=G3,G6,G1&VW=T.

[10] Jacobs JA. Gender inequality and higher education. Annu Rev Sociol. 1996;22: 153–85. 10.1146/annurev.soc.22.1.153

[11] Bellas ML, Toutkoushian RK. Faculty time allocations and research productivity: Gender, race and family effects. Rev High Educ. 1999;22(4): 367–90.

[12] Schuster JH, Finkelstein MJ. The American Faculty: The Restructuring of Academic Work and Careers Baltimore: The Johns Hopkins University Press; 2006.

[13] Van Arensbergen P, Hessels L, Van der Meulen B. Talent Centraal: Ontwikkeling en selectie van wetenschappers in Nederland The Hague: Rathenau Instituut; 2013.

[14] van den Brink M, Benschop Y. Gender practices in the construction of academic excellence: Sheep with five legs. Organization. 2012;19(4): 507–24.

[15] Prpić K. Gender and productivity differentials in science. Scientometrics. 2002;55(1): 27–58.

[16] Larivière V, Vignola-Gagné E, Villeneuve C, Gélinas P, Gingras Y. Sex differences in research funding, productivity and impact: an analysis of Quebec university professors. Scientometrics. 2011;87(3): 483–98. 10.1007/s11192-011-0369-y

[17] Van Arensbergen P. Talent Proof: Selection Processes in Research Funding and Careers. Doctoral dissertation, VU University, 2014. Available: http://hdl.handle.net/1871/51588.

[18] van den Besselaar P, Sandström U. Gender differences in research performance and its impact on careers: a longitudinal case study. Scientometrics. 2016;106(1): 143–62. 10.1007/s11192-015-1775-3 26798162 PMC4709377

[19] Bordons M, Morillo F, Fernández MT, Gómez I. One step further in the production of bibliometric indicators at the micro level: Differences by gender and professional category of scientists. Scientometrics. 2003;57(2): 159–73.

[20] Steinpreis RE, Anders KA, Ritzke D. The impact of gender on the review of the curricula vitae of job applicants and tenure candidates: A national empirical study. Sex Roles. 1999;41(7–8): 509–28.

[21] Ward ME. Gender and promotion in the academic profession. Scot J Polit Econ. 2001;48(3): 283–302.

[22] Austen S. Gender Differences in Academic Rank in Australian Universities. Australian Bulletin of Labour. 2004;30(2): 113–33.

[23] Cooray A, Verma R, Wright L. Does a gender disparity exist in academic rank? Evidence from an Australian university. Appl Econ. 2014;46(20): 2441–51.

[24] Van den Brink M, Brouns M, Waslander S. Does excellence have a gender?: A national research study on recruitment and selection procedures for professorial appointments in The Netherlands. Employee Relations. 2006;28(6): 523–39.

[25] Kaminski D, Geisler C. Survival Analysis of Faculty Retention in Science and Engineering by Gender. Science. 2012;335(6070): 864–6. 10.1126/science.1214844 22344445

[26] Ginther DK, Kahn S. Does Science Promote Women? Evidence from Academia 1973–2001 In: Freeman RB, Goroff DF, editors. Science and Engineering Careers in the United States. Chicago: University of Chicago; 2009 pp. 163–94.

[27] Ceci SJ, Williams WM. Understanding current causes of women's underrepresentation in science. P Natl Acad Sci USA. 2011;108(8): 3157–62. 10.1073/pnas.1014871108 21300892 PMC3044353

[28] European Research Council. Gender statistics; 2014.

[29] van der Lee R, Ellemers N. Gender contributes to personal research funding success in The Netherlands. P Natl Acad Sci USA. 2015;112(40): 12349–53. 10.1073/pnas.1510159112 26392544 PMC4603485

[30] Volker B, Steenbeek W. No evidence that gender contributes to personal research funding success in The Netherlands: A reaction to van der Lee and Ellemers. P Natl Acad Sci USA. 2015;112(51): E7036–E7. 10.1073/pnas.1519046112 26647179 PMC4697409

[31] Maas B, Korvorst M, van der Mooren F, Meijers R. Careers of Doctorate Holders in the Netherlands, 2014. The Hague/Heerlen: Statistics Netherlands; 2014.

[32] Bender KA, Heywood JS. Job satisfaction of the highly educated: The role of gender, academic tenure, and earnings. Scot J Polit Econ. 2006;53(2): 253–79.

[33] Bloch C, Graversen EK, Pedersen HS. Researcher mobility and sector career choices among doctorate holders. Res Evaluat. 2015;24(2): 171–80. 10.1093/reseval/rvv004

[34] Dever M, Boreham P, Western M, Haynes M, Kübler M, Laffan W, et al. Gender Differences in Early Post-PhD Employment in Australian Universities: The influence of PhD Experience on Women’s Academic Careers. Brisbane: The University of Queensland Social Research Centre (UQSRC); 2008.

[35] Fox MF, Stephan PE. Careers of young scientists: Preferences, prospects and realities by gender and field. Soc Stud Sci. 2001;31(1): 109–22.

[36] Sonneveld H, Yerkes M, Van de Schoot R. Ph.D. trajectories and labour market mobility: a survey of recent doctoral recipients at four universities in the Netherlands. Utrecht: Netherlands Centre for Graduate and Research Schools; 2010.

[37] Auriol L, Misu M, Freeman RA. Careers of Doctorate Holders: Analysis of Labour Market and Mobility Indicators. Paris: OECD; 2013.

[38] Van der Steeg M, Van der Wiel K, Wouterse B. Individual Returns to a PhD Education in the Netherlands: Income Differences between Masters and PhDs. The Hague: Centraal Planbureau, 2014.

[39] Netherlands Institute for Social Researh/Statistics Netherland. Emancipatiemonitor 2014. The Hague: Netherlands Institute for Social Research/Statistics Netherlands, 2014.

[40] Portegijs W, Cloïn M, Ooms I, Eggink E. Hoe het werkt met kinderen: Moeders over kinderopvang en werk The Hague: Sociaal Cultureel Planbureau; 2006.

[41] Waaijer CJF, Belder R, Sonneveld H, Van Bochove CA, Van der Weijden ICM. Survey on the Labour Market Position of PhD Graduates: Development of a Novel Questionnaire Leiden: Centre for Science and Technology Studies; 2015.

[42] OECD. Frascati Manual: Proposed Standard Practice for Surveys on Research and Experimental Development Paris: OECD; 2002.

[43] Deeltijdwerk. 2009 22 July [cited 18 November 2015]. Available: http://www.cbs.nl/nl-NL/menu/methoden/toelichtingen/alfabet/d/deeltijdwerk.htm.

[44] Demographic and socio-economic indicators, GDP per capita, PPP (current international $) [cited 29 October 2014]. Available: http://data.uis.unesco.org/Index.aspx?DataSetCode=DEMO_DS.

[45] Ministerie van Sociale Zaken en Werkgelegenheid. Monitor Arbeidsmarkt. The Hague: Ministerie van Sociale Zaken en Werkgelegenheid; 2014.

[46] Waaijer CJF, Belder R, Sonneveld H, van Bochove CA, van der Weijden ICM. Temporary contracts: Effect on job satisfaction and personal lives of recent PhD graduates. High Educ. 2016; In press.

[47] Mandel H. Up the Down Staircase: Women's Upward Mobility and the Wage Penalty for Occupational Feminization, 1970–2007. Soc Forces. 2013;91(4):1183–207.

[48] International Labour Organization. International Standard Classification of Occupations Geneva: ILO; 2008.

[49] De Kok JMP, De Jonge J, Tom M. Tijdsbesteding universitair wetenschappelijk personeel. Zoetermeer: EIM; 2007.

[50] Stephan P, Scellato G, Franzoni C. International Competition for PhDs and Postdoctoral Scholars: What Does (and Does Not) Matter. Atlanta: Andrew Young School of Policy Studies Research Paper Series; 2014.

[51] Baker M. Career confidence and gendered expectations of academic promotion. J Sociol. 2010;46(3): 317–34. 10.1177/1440783310371402

[52] Maes K, Gvozdanovic J, Buitendijk S, Rahm Hallberg I, Mantilleri B. Women, research and universities: excellence without gender bias Leuven: League of European Research Universities; 2012.

[53] van den Brink M, Benschop Y. Slaying the Seven-Headed Dragon: The Quest for Gender Change in Academia. Gender Work Organ. 2012;19(1): 71–92.

[54] Blickenstaff JC. Women and science careers: leaky pipeline or gender filter? Gender Educ. 2005;17(4): 369–86. 10.1080/09540250500145072

[55] Miller DI, Eagly AH, Linn MC. Women's Representation in Science Predicts National Gender-Science Stereotypes: Evidence From 66 Nations. J Educ Psychol. 2015;107(3): 631–44. 10.1037/edu0000005

[56] Statistics Netherlands. Emancipatiemonitor 2014: Meisjes kiezen steeds vaker voor techniek, meer vrouwen dan eerst in topfunctie. 2014 Dec 16 [cited 21 October 2015]. Available: http://www.cbs.nl/nl-NL/menu/themas/dossiers/vrouwen-en-mannen/publicaties/artikelen/archief/2014/2014-080b-pb.htm.

[57] OECD Employment Database, Female and male share in part-time employment, as a proportion of total employment, 1994–2010. 2012 [cited 29 October 2015]. Available: http://skills.oecd.org/supplyskills/documents/22bfemaleandmaleshareinpart-timeemployment.html.

[58] Bosch N, Van Ours J, Van der Klaauw B. Female part-time work in the Netherlands. 2009 September 5 [cited 2015 29 October]. Available: http://www.voxeu.org/article/why-dutch-women-work-part-time.

[59] Visser J. The first part-time economy in the world: a model to be followed? J Eur Soc Policy. 2002;12(1): 23–42.

